# Wireless Sensor Network-Based Greenhouse Environment Monitoring and Automatic Control System for Dew Condensation Prevention

**DOI:** 10.3390/s110403640

**Published:** 2011-03-25

**Authors:** Dae-Heon Park, Jang-Woo Park

**Affiliations:** Department of Information and Communication Engineering, National University of Sunchon, Maegok 315, Suncheon, Jeonnam, Korea; E-Mail: dhpark@scnu.ac.kr

**Keywords:** greenhouse, leaf sensor, dew point, dew-condensation prevention, sensor network

## Abstract

Dew condensation on the leaf surface of greenhouse crops can promote diseases caused by fungus and bacteria, affecting the growth of the crops. In this paper, we present a WSN (Wireless Sensor Network)-based automatic monitoring system to prevent dew condensation in a greenhouse environment. The system is composed of sensor nodes for collecting data, base nodes for processing collected data, relay nodes for driving devices for adjusting the environment inside greenhouse and an environment server for data storage and processing. Using the Barenbrug formula for calculating the dew point on the leaves, this system is realized to prevent dew condensation phenomena on the crop’s surface acting as an important element for prevention of diseases infections. We also constructed a physical model resembling the typical greenhouse in order to verify the performance of our system with regard to dew condensation control.

## 1. Introduction

Environmentally-friendly high-quality agriculture has been investigated in order to improve the farming practices in greenhouses. Recent developments in the field of wireless sensor networks as well as miniaturization of the sensor nodes has allowed precision agriculture to emerge. Precision agriculture concentrates on providing the means for harvest information, work management and growth information [1,2].

The National Institute of Rural Engineering conducts studies on measuring biological and environmental situations and the crop monitoring systems. The research topics are the effects of dehumidification on air temperature and relative humidity in *Phalaenopsis* greenhouses [3]. Research for improving agricultural productivity by introducing sensor network technologies to the field of agriculture is in progress. Stipanicev studied network embedded greenhouse monitoring and control based on a TINI embedded Web server unit which gathers and routes data from local sensor/actuator networks to a global network-Internet [4]. Zhou *et al.* proposed an architecture and application of a ZigBee-based mesh network combined with event-based control techniques [5]. Ahonen monitored the environment of a greenhouse using a WSN and assessed the network using collected data [6]. Kang *et al.* developed an automatic greenhouse environment monitoring and control system. They studied the development of environmental monitoring sensor nodes and a monitoring system in greenhouses [7]. Nielsen’s work deals with the development and testing of computer algorithms designed to spread the energy demand of greenhouses, and he showed that without reduction in plant quality, it is possible to avoid energy consumption peaks in the morning and in the evening hours [8]. Candido *et al.* described an embedded real-time system for climate control in a complex greenhouse [9]. Li *et al.* designed a remote monitoring system for the greenhouse environment. They deal with the software of the embedded web remote monitoring system for greenhouse environments [10]. Sun *et al.* designed an embedded database system for temperature and humidity control in the greenhouse [11]. Pawloski *et al.* proposed event-based systems on wireless sensor networks for climate control in the greenhouse [12]. The Rinnovando group is working with agricultural experts on a short-term deployment of a wireless sensor network in a tomato greenhouse in the South of Italy [13].

Greenhouse environment monitoring and control is essential to improve productivity through prevention of diseases in the crops. The dew condensation phenomenon occurs in the greenhouse when the dew point temperature is higher than the temperature of crops, and it is deeply related to relative humidity. Especially, too close to sunrise with the high humidity at daybreak or when humidity inside a greenhouse is too high, the temperature inside a greenhouse gets to rise rapidly but the temperatures of crops rise slowly. Thus the huge difference between the environmental temperature and the crop temperature causes the dew condensation phenomenon to occur. The dewdrops on the leaves can play an important role as a culture medium through which diseases such as *Leveillula taurica Arnaud* may proliferate. Forecasting dewdrop generation and removing the dewdrops are important for growing crops in the greenhouse. We propose herein a dew condensation prevention system that is able to forecast the conditions of dew condensation generation and remove the dewdrops based on ambient temperature and humidity data, as well as leaf temperature values.

## 2. Automatic Dew-Condensation Control System

This paper proposes an automatic dew condensation control system for maintaining proper humidity and temperature in crop cultivation and preventing the proliferation of diseases that can result from formation of dew on a leaf surface. The temperature of the dew point is calculated with a Barenbrug formula [1] at an environmental server using ambient temperature and relative humidity as well as leaf temperature data collected by sensors.

### 2.1. Building Blocks of Automatic Dew Condensation Control System

**Figure 1.**
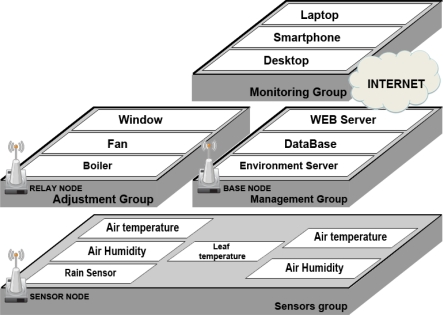
Overall System Diagram.

Figure 1 is overall system diagram of our automatic dew condensation control system. It is composed of a sensor unit, a managing unit and monitoring unit. The sensor nodes installed inside a greenhouse allows the ambient temperature and humidity sensor and a leaf temperature sensor to collect environmental data. The collected data are transmitted wirelessly to the base nodes, and the base nodes transmit data from each sensor to the environment server through serial communications. Sensor nodes and base nodes are deployed inside the green house to make up the wireless sensor network. The environment server manages the entire system, so it has to receive the data from the relay nodes, process it and send out the decision and control signals. The environmental server can also estimate the dew point conditions with the Barenbrug formula. Together the data collected from a sensor and the decision and control signals processed and transmitted by the environmental server can provide the environmental settings that a facility manager wants. The relay nodes can run devices such as ventilation fans, windows and internal circulating fans, which are able to adjust the environment of the greenhouse, through control signals sent from the greenhouse environment server. The data processed in the environment server are stored in a database, and the greenhouse state information is monitored and controlled from some platform (laptop PC, PDA, desktop PC) being used by a manager through the Internet or a web server.

### 2.2. Sensor Nodes Design

Sensor nodes designed in this system [14] receive measured data from temperature, humidity, leaf temperature and rain sensors, process the data with a microprocessor (MSP430 MCU) and transmit the data to a PC and relay nodes using a transceiver (CC2420 RF chip). Nodes and sensors are designed to be separate from each other to minimize the effect of heat emitted from nodes on sensors. The MSP430 microprocessor has a 16 bit RISC structure and has 48 KB of program memory and 10 KB RAM, which can handle multiple sensor data simultaneously with high speed. The CC2420 transceiver is a RF chip supporting Zigbee that works in the 2,400∼2,483.5 MHz frequency band. Communication is made by DDDS method, supporting O-QPSK modulation and 250 Kbps data rate, which makes low-power real-time wireless communication possible [15].

**Figure 2.**
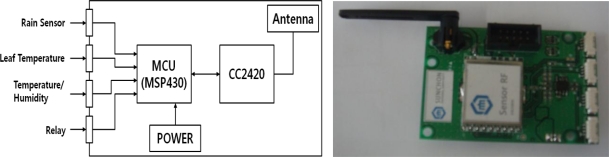
Block diagram of the sensor node and the as-manufactured sensor node.

Figure 2 shows a block diagram of sensor node and the as-manufactured sensor node. It is composed of the MSP430 unit for data processing, CC2420 for data wireless transmission, environmental sensor, power supply and antenna.

**Figure 3.**
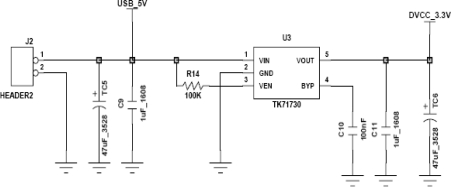
Power supply circuit diagram.

Figure 3 shows the power supply circuit diagram. The power supply for the sensor node uses a 3.6 V battery. A TK71730 LDO is used to supply stable power to the nodes [16]. The LDO consists of a pass transistor and error amplifier. The pass transistor is composed of a PNP TR or PMOS, and works as a voltage-controlled current source. The error amplifier receives feedback from the output to maintain a stable voltage, even with input voltage or output load changes.

**Figure 4.**
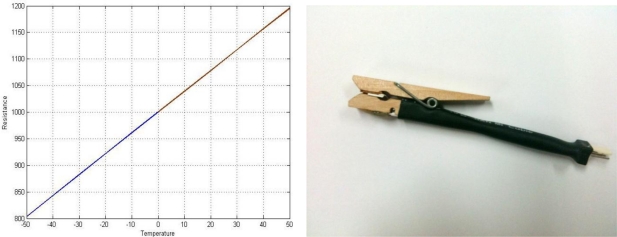
Characteristic leaf temperature curve and sensor.

Careful attention is needed for sensor selection because of its importance in measuring and controlling the environment affecting crop growth in practical greenhouse cultivation. It is important to select sensors which can withstand the high temperatures and high humidity of the greenhouse environment and have high sensitivity and reliability in a suitable range for crop cultivation. The SHT71 is used as temperature/humidity sensor [17]. It has a combined temperature sensor and a humidity sensor. It uses a low power supply of 2.4 V∼5.5 V, and has a low power consumption of 28 μA. The temperature measurement range is from −40 °C to 120 °C and the measurement has 0.5% tolerance, and the humidity measurement range is from 0% to 100%, with 3.5% tolerance. A non-contact infrared sensor was used as the leaf temperature sensor, but as its current variation when measuring the temperature of a leaf is very small, making it difficult to measure the leaf’s temperature accurately. A contact leaf temperature sensor developed by PhyTech Ltd. is available, but it has the disadvantages of being very expensive and difficult to buy in Korea. A leaf temperature sensor using RTD (Resistance Temperature Detector) has been designed to accurately measure the temperature of a leaf [18] by converting the resistance value change of a RTD (PT1000) sensor according to temperature change in a leaf into a temperature value, while directly contacting the RTD (PT1000) sensor on a leaf surface of crops using the RTD (PT1000) sensor. The RTD (PT1000) sensor can measure leaf temperature in the range between −50 °C and 50 °C. The RTD (PT1000) sensor has ±0.3 °C tolerance. Figure 4 shows a characteristic temperature graph of a PT1000 sensor and the designed leaf temperature sensor. The temperature characteristic graph was plotted with Matlab using the following numerical formula:

(1) $$-50{}^{\circ}C∼0{}^{\circ}C:R(t)=R0\left(1+\mathrm{At}+{\mathrm{Bt}}^{2}+C(t-100)\times t^{3}\right)$$

(2) $$0{}^{\circ}C∼50{}^{\circ}C:R(t)=R0\left(1+\mathrm{At}+{\mathrm{Bt}}^{2}\right)$$

where:

A: 3.9083 × 10^−3^ °C^−1^, B: −5.775 × 10^−7^ °C^−2^,
C: −4.183 × 10^−12^ °C^−4^
R0: resistance value in ohm at 0 °C
t: temperature

**Figure 5.**
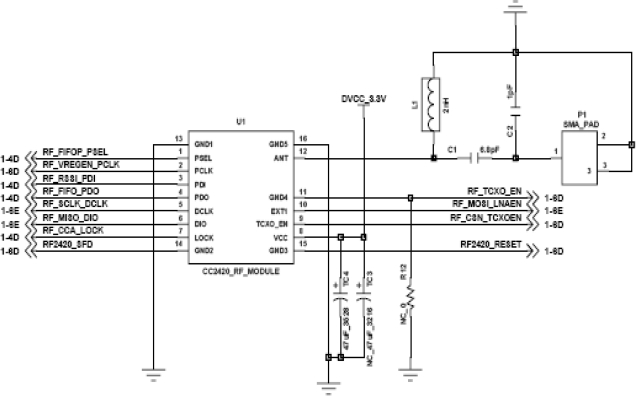
CC2420 circuit diagram.

Figure 5 shows wireless circuit diagram. The Wireless Communication Module (CC2420, Chipcon) is a 2.4 GHz Zigbee chip, which is adequate for low power and low cost sensor network construction. It uses O-QPSK modulation with the DSSS (Direct Sequence Spread Spectrum) method and Half Sine Pulse [19]. It has a maximum transmission speed of 250 Kbps. It has four serial ports -SI, SO, SCLK, and CSn-, 33 configuration/status 16-bit register, 15 command storage register, and two 8-bit transmit/receive FIFO registers.

**Figure 6.**
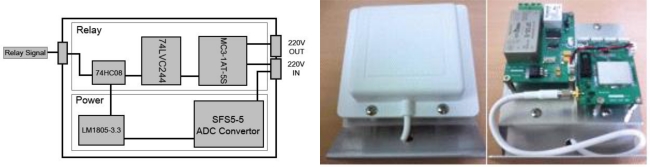
Relay node block diagram and manufactured relay node.

The relay node receives operation signals from the base node when the greenhouse environment data exceeds the preset threshold value, and provides power to the corresponding equipment. 220 V power is applied to the relay node, and the node gets 5 V output voltage through a SFS5-5 converter and gets 3.3 V node power through an LM1085-3.3 regulator circuit. It operates windows, ventilators, and heaters by providing the power supply with a signal connection to a HC3-1AT-5S relay made by Handouk Co. It sends a signal to the relay through the 74HC08 AND gate when it receives an operational signal from a sensor node using a 2.4 GHz dipolar antenna, and through a 74LVC244 cctal buffer when both POWER and the operational signal are high. Figure 6 shows the designed relay node block diagram and its actual picture.

It is designed and realized with a high performance HG2409PCL-SM 2.4 GHz antenna connected to the sensor node for receiving smooth relay operation signals. A stable power supply is essential for sensor nodes. For the relay node, it receives AC 220 V, converts it to DC 5 V output using a SFS5_5 chip, and provides stable 3.3 V power to nodes using a LM1085-3.3 chip [20]. It transmits signals to the 74LVC244 when both a 3.3 V power supply and a signal from the sensor node are received as input through the 74HC08 AND gate. 74LVC244 is an octal buffer and has the function of transmitting a signal when it receives a signal, and it protects from reverse flow currents. Signals, which pass through the octal buffer are sent to the HC3-1AT-5S relay to provide the power supply. The HC3 relay is lighter than normal relays, has smooth structure, and has very sensitive characteristics.

### 2.3. Dew Point

The ambient temperature and humidity as well as leaf temperature values collected with environmental sensor nodes are used to calculate the dew point. The collected data are substituted for the dew point formula of Barenbrug. This equation has an error value of ±0.4 °C [1] and it is valid for:

(3) $$\begin{array}{l} 0{}^{\circ}C<T<60{}^{\circ}C\\ \;\;1\%<\text{RH}<100\%\\ 0{}^{\circ}C<\text{Td}<50{}^{\circ}C \end{array}$$

where:

T: Temperature in degrees Celsius
RH: Relative humidity (4)
Td: Dew point temperature

The equation is:

(5) $$T_{d}=\frac{b\cdot \alpha (T,\mathrm{RH})}{a-\alpha (T,\mathrm{RH})}$$

where:

(6) $$\alpha (T,\mathrm{RH})=\frac{a\cdot T}{b+T}+\text{ln}(\mathrm{RH}/100)$$

and a = 17.27, b = 237.3 °C (a, b is constant)

### 2.4. Flowchart of Automatic Dew Condensation Control System

**Figure 7.**
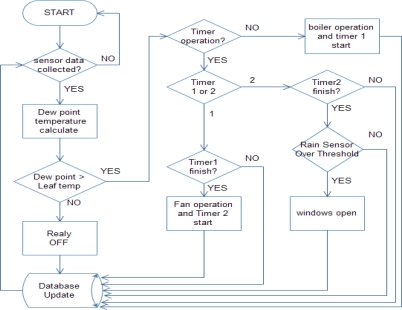
Automatic dew condensation control system flowchart.

Figure 7 shows a flowchart of the proposed automatic dew condensation control system. The environment server calculates the dew point temperature and compares it with the leaf temperature based on data collected from a sensor node. At this time, when the dew point temperature is higher than the leaf temperature, occurrence of a favorable dew condensation environment will be judged. When we use a timer, the experimental operating process flow of the automatic dew condensation control system is as follows:

1. The dew point temperature is calculated with the collected information.
2. The calculated dew point temperature is compared with the leaf temperature.
3. If the leaf temperature is lower than the dew point temperature, judge if the timer is operated. Each timer has a value set by the administrator, which is the operation time of actuators.
4. If a timer1 and a timer2 are not operated in step 3, operate the internal circulating fan with an actuator.
5. When the timer1 and the timer2 are operated in step 3, judge which timer is operating.
6. When operation of the timer1 is complete in step 3, operate timer2 and an external circulating fan.
7. When it is judged to rain with a rain sensor mounted outside the greenhouse, don’t open its window.
8. When step 7 does not indicate rain, adjust temperature and humidity inside the greenhouse by opening the windows.
9. The device operation situations and all data in the above total processes are stored in a database.

## 3. System Realization

**Figure 8.**
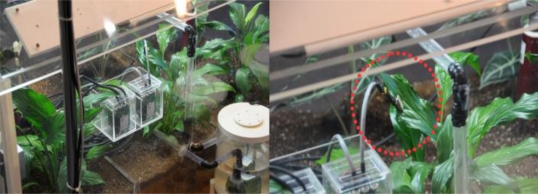
A model greenhouse.

The performance of the automatic dew condensation control system was verified by making a model similar to the internal environment of an actual greenhouse as shown in Figure 8.

**Figure 9.**
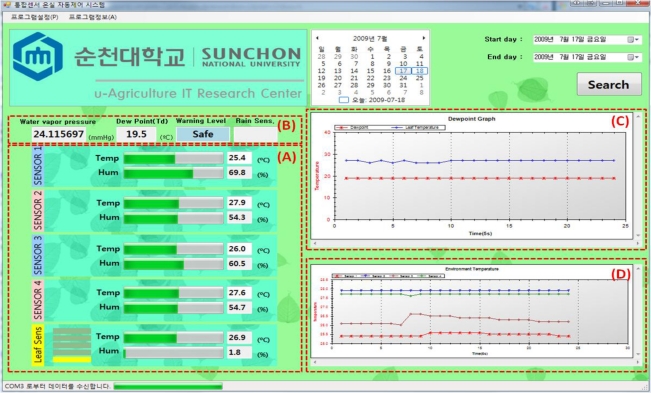
GUI Screen.

Figure 9 is a GUI screen to show the greenhouse environmental information obtained and processed with the automatic dew condensation control system. The information collected through an environmental sensor mounted in a greenhouse model is stored in the database and the GUI (Graphical User Interface) can provide the greenhouse condition details to a manager. The dewdrop generation conditions are monitored with sensor nodes and judged by calculating the dew point from the collected data. Data are collected at 5 second interval from each sensor installed in the greenhouse. There are graphs showing the leaf temperature and the dew point temperature and an ambient temperature graph showing temperature changes of the greenhouse environment according to each sensor node in the GUI screen. Window (A) shows the collected data from environment sensors 1–4 and the leaf sensor, and (B) show values calculated using the environment data from each sensor and the leaf sensor; (B) includes the water vapor pressure, dew point, warning level and check for rain outside. Window (C) compares the dew point temperature and leaf temperature, (D) shows the temperature graph of each environment sensor. A manager can directly change environmental setting parameters of the greenhouse through the GUI and control devices of the greenhouse.

**Figure 10.**
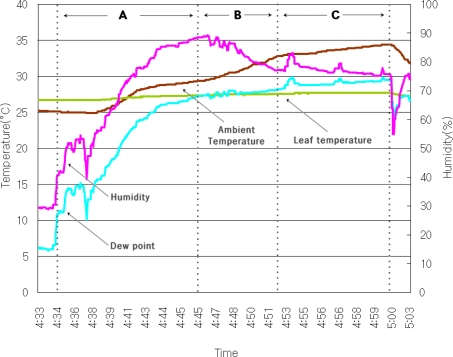
Experimental results.

Figure 10 shows the results obtained from the the greenhouse model equipped with the automatic dew condensation control system. The experimental environment was as follows: an internal circulating fan, ventilating fan, side windows, ceiling, warm air circulator and humidifier were installed in the greenhouse model. The change in Part A of Figure 10 shows the greenhouse condition when we create conditions for dew condensation. We maintain an internal humidity of more than 80% and cause the temperature to rapidly raise using the heater. The experimental results of the automatic dew condensation control system in the greenhouse environment are as follows.

1. In part B of Figure 10, it can be seen that when the dew point temperature is higher than the leaf temperature, the humidity gets lower by operation of an internal circulating fan’s actuator.
2. Because the dew point temperature of the greenhouse is higher than the leaf temperature even after operation of the internal circulating fan explained above, the humidity and temperature of the greenhouse were continuously adjusted by operating a ventilating fan.
3. It can be seen that because the dew point temperature is higher than the leaf temperature even after operation of the above step, the automatic dew condensation control system rapidly lowers the temperature and humidity by opening side windows and ceilings like in part C of Figure 10.

It shows our system can continuously control the inside conditions of the greenhouse through the devices placed in the greenhouse. The automatic dew condensation control system proposed in this paper can forecast and prevent dew condensation by controlling the conditions of formation of dewdrops in the greenhouse environment.

## 4. Conclusions and Future Work

In this paper, we have designed and implemented a system that can understand the greenhouse environment and the state of crops by using sensors and optimize crop growth conditions with emphasis on the dew point condition. An automatic dew condensation control system combined with a WSN was realized, which utilizes the dew point condition to prevent the dew condensation phenomenon on the leaf surfaces of crops that is believed to be decisive in the outbreak of crop diseases. Also, a model similar to an actual greenhouse environment was made to verify the performance of the system presented and the model was operated and monitored by applying the automatic dew condensation control system. It can also cope with exceptional situations by providing the greenhouse environment and information about a device’s operating state to users every certain time. The topic to be researched in the future is the optimal sensor deployment in a real greenhouse for the automatic dew condensation control system. To apply the automatic dew condensation control system to an actual greenhouse environment, we will have to gather more data about the real conditions and refine our system. Additionally, the building blocks composing the automatic dew condensation control system should be extended so that it can be applied to various situations that can occur in the actual greenhouse environment.
